# Supplementary material for: Internet search patterns reveal clinical course of COVID-19 disease progression and pandemic spread across 32 countries
Source: NPJ Digit Med. 2021 Feb 11;4:22. doi: 10.1038/s41746-021-00396-6 (PMC7878474; doi:10.1038/s41746-021-00396-6)
Supplement: Supplementary file 1 — Supplementary Information [file 41746_2021_396_MOESM1_ESM.pdf]

Supplementary Information

Supplementary Table 1. Comprehensive list of lags between each of the search terms and COVID-19 deaths, for each of the 32 countries.

Country	Coronavirus symptoms	Coronavirus test	Fever	Cough	Runny nose	Dry cough	Sore throat	Chills	Shortness of breath
**Argentina**	24	19	20	21	24	23	22	26	21
**Australia**	23	22	20	18	20	19	19	16	20
**Belgium**	25	26	26	24	27	24	25	29	24
**Brazil**	21	28	25	25	25	25	25	22	18
**Canada**	30	30	28	27	29	28	28	22	27
**Switzerland**	25	20	24	25	17	19	23	13	15
**Chile**	24	21	22	22	23	21	23	24	20
**Colombia**	24	21	23	21	23	21	21	25	25
**Germany**	30	30	28	26	16	23	30	22	18
**Spain**	18	17	15	15	17	15	14	18	13
**France**	21	22	20	19	19	19	19	15	10
**U.K.**	27	23	23	22	23	22	22	22	22
**Guatemala**	29	22	29	30	25	22	26	29	13
**Hungary**	26	27	26	30	24	30	24	22	26
**Indonesia**	25	23	20	19	25	19	20	14	19

**Ireland**	30	28	26	25	29	26	25	25	25
**India**	28	24	20	19	25	19	27	17	17
**Iran**	25	22	20	21	28	21	20	16	17
**Italy**	23	27	18	24	8	19	20	12	11
**Mexico**	28	26	26	24	22	27	26	27	21
**Malaysia**	13	12	12	12	0	11	6	20	4
**Nigeria**	25	21	23	22	19	23	20	21	17
**Netherlands**	30	16	19	20	19	19	20	17	17
**Poland**	27	25	30	26	30	25	25	22	18
**Saudi Arabia**	23	23	28	23	14	21	3	20	10
**Sweden**	30	23	30	24	21	21	25	12	22
**Turkey**	21	21	16	19	23	20	18	14	18
**United States**	30	28	25	25	27	25	24	22	25
**Uruguay**	18	18	19	16	18	19	17	18	7
**Venezuela**	16	15	12	15	18	12	16	9	8
**South Africa**	30	30	23	29	29	21	22	13	18
**Zimbabwe**	15	13	13	12	5	7	12	11	30

Supplementary Table 2. Translations used for each search term in each country.

Part I. Columns 1-6:

Country	fever	dry cough	coronavirus test	coronavirus symptoms	cough
**Argentina**	fiebre	tos seca	prueba del coronavirus	sintomas del coronavirus	tos
**Australia**	fever	dry cough	coronavirus test	coronavirus symptoms	cough
**Belgium**	koorts	droge hoest	coronatest	coronasymptomen	hoest
**Brazil**	febre	tosse seca	exame de coronavirus	sintomas do coronavírus	tosse
**Canada**	fever	dry cough	coronavirus test	coronavirus symptoms	cough
**Switzerland**	fieber	trockener husten	coronavirus test	coronavirus symptome	husten
**Chile**	fiebre	tos seca	prueba del coronavirus	sintomas del coronavirus	tos
**Colombia**	fiebre	tos seca	prueba del coronavirus	sintomas del coronavirus	tos
**Germany**	fieber	trockener husten	coronavirus test	coronavirus symptome	husten
**Spain**	fiebre	tos seca	prueba del coronavirus	sintomas del coronavirus	tos
**France**	fièvre	toux sèche	test du coronavirus	symptômes du coronavirus	toux
**U.K.**	fever	dry cough	coronavirus test	coronavirus symptoms	cough
**Guatemala**	fiebre	tos seca	prueba del coronavirus	sintomas del coronavirus	tos
**Hungary**	láz	száraz köhögés	koronavírus teszt	koronavírus tünetei	köhögés
**Indonesia**	demam	batuk kering	uji coronavirus	gejala virus Corona	batuk
**Ireland**	fever	dry cough	coronavirus test	coronavirus symptoms	cough
**India**	fever	dry cough	coronavirus test	coronavirus symptoms	cough

**Iran**	تب	سرفه خشک	آزمایش کرونا	علائم کرونا	سرفه
**Italy**	febbre	tosse secca	test per il coronavirus	sintomi del coronavirus	tosse
**Mexico**	fiebre	tos seca	prueba del coronavirus	sintomas del coronavirus	tos
**Malaysia**	demam	batuk kering	Ujian coronavirus	simptom koronavirus	batuk
**Nigeria**	fever	dry cough	coronavirus test	coronavirus symptoms	cough
**Netherlands**	koorts	droge hoest	coronatest	coronavirus symptomen	hoest
**Poland**	gorączka	suchy kaszel	test na koronawirusa	objawy koronawirusa	kaszel
**Saudi Arabia**	حمى	سعال جاف	فيروس كورونا فحص	أعراض كورونا	سعال
**Sweden**	feber	rethosta	coronavirus prov	coronavirus symptom	hosta
**Turkey**	ateş	Kuru öksürük	coronavirüs testi	koronavirüs belirtileri	öksürük
**United States**	fever	dry cough	coronavirus test	coronavirus symptoms	cough
**Uruguay**	fiebre	tos seca	prueba de coronavirus	sintomas del coronavirus	tos
**Venezuela**	fiebre	tos seca	prueba de coronavirus	sintomas del coronavirus	tos
**South Africa**	fever	dry cough	coronavirus test	coronavirus symptoms	cough
**Zimbabwe**	fever	dry cough	coronavirus test	coronavirus symptoms	cough

Part II. Columns 7-11:

Country	runny nose	shortness of breath	chills	sore throat
**Argentina**	rinorrea	dificultad para respirar	resfriado	dolor de garganta
**Australia**	runny nose	shortness of breath	chills	sore throat
**Belgium**	loopneus	kortademigheid	koude rillingen	keelpijn
**Brazil**	coriza	falta de ar	calafrios	dor de garganta
**Canada**	runny nose	shortness of breath	chills	sore throat
**Switzerland**	nez qui coule	kurzatmigkeit	frissons	mal de gorge
**Chile**	rinorrea	dificultad para respirar	resfriado	dolor de garganta
**Colombia**	rinorrea	dificultad para respirar	gripa	dolor de garganta
**Germany**	laufende Nase	kurzatmigkeit	schüttelfrost	Halsschmerzen
**Spain**	rinorrea	dificultad para respirar	resfriado	dolor de garganta
**France**	nez qui coule	essoufflement	frissons	mal de gorge
**U.K.**	runny nose	shortness of breath	chills	sore throat
**Guatemala**	rinorrea	dificultad para respirar	resfriado	dolor de garganta
**Hungary**	orrfolyás	légszomj	hidegrázás	Torokfájás
**Indonesia**	pilek	sesak napas	panas dingin	sakit tenggorokan
**Ireland**	runny nose	shortness of breath	chills	sore throat
**India**	runny nose	shortness of breath	chills	sore throat
**Iran**	آبریزش بینی	تنگی نفس	لرز	گلو درد

**Italy**	rinorrea	respiro corto	brividi	mal di gola
**Mexico**	rinorrea	dificultad para respirar	resfriado	dolor de garganta
**Malaysia**	hidung berair	sesak nafas	menggigil	sakit tekak
**Nigeria**	runny nose	shortness of breath	chills	sore throat
**Netherlands**	loopneus	kortademigheid	rillingen	keelpijn
**Poland**	Katar	duszność	dreszcze	ból gardła
**Saudi Arabia**	سيلان الأنف	1.ضيق في التنفس	1.قشعريرة	احتقان في الحلق
**Sweden**	rinnande näsa	andnöd	frossa	ont i halsen
**Turkey**	burun akıntısı	nefes darlığı	titreme	boğaz ağrısı
**United States**	runny nose	shortness of breath	chills	sore throat
**Uruguay**	rinorrea	dificultad para respirar	resfriado	dolor de garganta
**Venezuela**	rinorrea	dificultad para respirar	resfriado	dolor de garganta
**South Africa**	runny nose	shortness of breath	chills	sore throat
**Zimbabwe**	runny nose	shortness of breath	chills	sore throat
